# A review on ZnO-based electrical biosensors for cardiac biomarker detection

**DOI:** 10.4155/fsoa-2017-0006

**Published:** 2017-06-07

**Authors:** Nandhinee R Shanmugam, Sriram Muthukumar, Shalini Prasad

**Affiliations:** 1 Department of Bioengineering, University of Texas at Dallas, Richardson, TX 75080, USA; 2 Enlisense LLC, Allen, TX 75013, USA

**Keywords:** biomarker, biosensor, disease diagnosis, electrical detection, POC, point-of-care, zinc oxide

## Abstract

Over the past few decades zinc oxide (ZnO)-based thin films and nanostructures have shown unprecedented performance in a wide range of applications. In particular, owing to high isoelectric point, biocompatibility and other multifunctional characteristics, ZnO has extensively been studied as a transduction material for biosensor development. The fascinating properties of ZnO help retain biological activity of the immobilized biomolecule and help in achieving enhanced sensing performance. As a consequence of recent advancements in this multidisciplinary field, diagnostic biosensors are expanding beyond traditional clinical labs to point-of-care and home settings. Label-free electrical detection of biomarkers has been demonstrated using ZnO-sensing platforms. In this review we highlight the characteristics of ZnO that enable realization of its use in development of point-of-care biosensors toward disease diagnosis, in particular cardiovascular diseases.

## Lay abstract

The goal of this review is to discuss the features of ZnO in enhancing the sensitivity and selectivity of biomarker detection toward the development of point-of-care diagnostic devices. Basic biosensor design features and fabrication of ZnO nanostructures, strategies for surface modifications and its application toward disease diagnosis are discussed.

Biosensors and bioanalytical devices are integrated devices that represent ingenious developments in the realm of early management of diseases. Design and development of such analytical devices harness the advantages of multidisciplinary fields such as engineering, chemistry and biology. The basic concept behind the design of these complex bioanalytical devices is their capability to deliver a measurable output signal response for either diagnostic or therapeutic purposes when a biological recognition element interacts with the transducing surface of the sensor. A biological recognition element, commonly known as a biomarker, is usually a biomolecule which represents the pathophysiological condition such as irregularities in cellular regulatory functions, pathological responses or intervention to any therapeutic drugs. Biomarkers are usually detected by employing another biological element known as bioreceptor (e.g., enzymes, nucleic acids, antibodies and so on) immobilized on to surface of transducer, which are sensitive to target analyte as shown in Figure 1. Successful strategies for objectively measuring these biomarkers include screening the complex body fluids for expression of any proteins, DNA or RNA expression profiles, circulating tumor cells, lipids, metabolites, etc. [1]. Qualitative and quantitative determination of these biomarker concentrations in biological fluids are used to differentiate healthy versus diseased state. Therefore, biomarkers have been identified as specific cues for disease diagnosis and are useful in therapeutic management of number of diseases [1].

**Figure 1.**
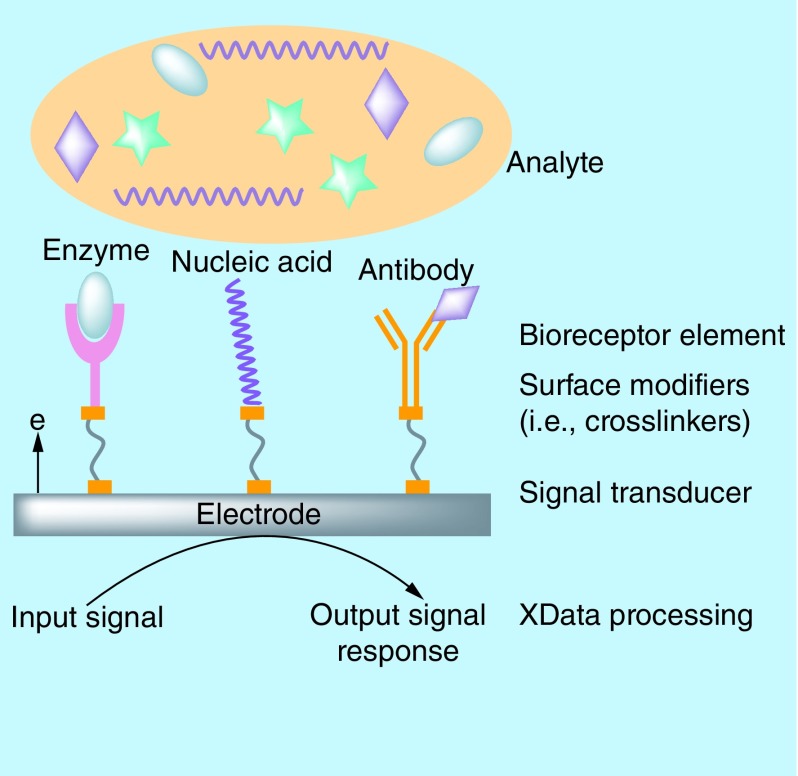
**General schematic of a biosensor.** Classified based on immobilized biological elements as enzyme, nucleic acid or affinity biosensors.

Illustration of the ‘enzyme electrode’ by Leland C Clark in 1962 marked the rise of a new era in healthcare diagnostics [2]. Since then, various studies have been conducted to enhance sensing performance, which has led to significant technological advancements over the past decades. Today, biosensors industry has witnessed tremendous growth with annual turnover anticipated at US$22.68 billion by 2020 [3]. The biosensor industry comprises two key categories of bioanalytical devices: traditional practices which involve testing in centralized clinical laboratories where sample processing, expensive equipment for analysis, need for trained laboratory personnel and higher turnaround time put significant cost and time burden on patients [4,5] and portable, inexpensive and easy-to-use point-of-care (POC) devices for testing in both clinical and nonclinical surroundings [6,7].

POC and rapid quantification of biomarkers can offer crucial information in various facets of disease detection, monitoring and analysis [6]. Currently, design and development of POC biosensors are one of the attractive sectors in a wide range of healthcare applications as it meets the immediate needs of a patient. POC testing aids patients and clinicians to perform testing remotely at the site of patient care and provides essential data almost instantaneously [7]. For diseases of which timely treatment highly impacts the quality and life of patients, POC testing has potential to improve therapeutic decisions. Thus, POC testing is more preferred as these analytical devices can significantly improve the healthcare efficiency and outcome, particularly in low-income countries [6]. The stringent requirements of POC devices are that they should be portable, reliable and testable by nontrained personnel. The POC biosensor market has evolved since the successful commercialization of glucose monitors and further technological advancements that are geared toward reducing the cost burden on patients. Today, POC diagnostic devices contribute to approximately 57% of overall biosensors market [3].

The growing prevalence of diseases and increasing demand for sophisticated healthcare infrastructure, as well as need for rapid and reliable disease detection have fueled biosensor research. Advancements in nanotechnology and biotechnology have set the stage for exciting possibilities to help address challenges in conventional biosensor design. The fundamental challenges in biomarker detection have been in achieving; ultrasensitivity, that is, detection of target biomarkers at relatively low concentrations (typically in lower pg/ml), and selectivity, that is, ability to distinguish target biomarker from other biomolecules present in sample biological fluid. The other challenges include low signal-to-noise ratio of readout mechanism, lower detection limits, low cost, minimal sample volume, long-term monitoring and fast and accurate detection [8]. Miniaturized and disposable biosensor chips that can perform these tasks can have significant impact in improving the quality of patient's life [9].

## Biosensor design concepts

Design of optimal biosensor devices requires a deeper understanding of both the biosensing concept in general as well as the targeted performance metrics. Miniaturization of biosensor devices is currently trending which has been made possible through advancements in nanofabrication methodologies. Miniaturization helps realize detection of multiple target biomarkers on the same platform and in batch manufacturing at very low cost, thus increasing the overall throughput. However, the transduction mechanism plays a significant role in development of miniaturized and integrated biosensors. Electrical/electrochemical transduction of physiochemical changes are simple and robust making miniaturization possible with limited resources, and unlike in optical biosensors, electrical transduction helps reduce signal interference from other biological molecules [10,11]. However, sensitivity and selectivity of biomarker detection are two important drivers for a successful establishment of these bioanalytical devices in the global healthcare market.

Synergy is the key to developing a perfect POC biosensor addressing the fundamental challenges with sensitivity, selectivity, stability and most importantly biocompatibility of the chosen material. Recent advances in nanofabrication have widened the research on new material synthesis, microfluidic design and understanding the biochemical interactions, all of which can be incorporated with electronics into a portable, miniaturized handheld POC device [7]. Nanomaterials are preferred over bulk materials owing to the surface and quantum confinement that they tend to offer. The nanotextured surfaces offer increased surface-area-to-volume ratio with increase in the sensitivity for detection of target analytes in their ambient environment. However, ambient tissue or fluid has a number of different analytes present that would interfere with specific detection [12]. Hence, another key element to be considered during biosensor design is the specificity/selectivity for the target analyte; where surface functionalization becomes very critical. Thus, the design process proceeds iteratively; selection of substrate materials as a transducing platform for building biointerfaces, surface modification of substrates to understand the immobilization strategies, optimization of physicochemical surface to obtain detectable real-time signal with reduced signal-to-noise ratio and, finally, systematic integration to build the most intelligent and smart biosensor [12,13].

A criterion for material selection is application specific, which includes choosing among metals/metal oxides, polymers/ceramics, hydrogels, etc. [10]. Polymer-based materials lack chemical moieties for surface functionalization, hence requires extensive pretreatment strategies. However, the complexity of these procedures interfere with the polymer surface altering its properties resulting in nonspecific interaction of biomolecules [14]. Hence, the most favorable material for biosensor design with specific functionalities is metal/metal oxides due to their well-known and transferrable surface chemistry approach for immobilization of biomolecules [15]. Recent advances and developments in nanofabrication techniques have potential applications in manufacturing nanomaterials for a wide range of applications. Nanomaterials such as nanoparticles, nanorods, nanowires, etc., are synthesized and employed to effectively improve the performance characteristics of a biosensor [13,15,16]. While the purpose for the use of an electrode remains the same, it is the strategy to tune the properties of electrodes that has sparked attention from researchers worldwide. Modification of electrode surfaces provides an opportunity to control and tune the performance of nanobiosensor platforms such as confer selectivity, resist fouling, eliminate nonspecific interactions and thus improve the S/N ratio [17]. Tailoring the electrode surface via nanostructuring provides enhanced charge transport and steady-state diffusion in electrochemical biosensors.

The surface topography of material used for transducing the physical or chemical changes due to biomolecular binding is a key feature in biosensor design. Surface topography can be altered by creating nanostructures specifically for size matching with the biomolecules. Nanotextured surfaces have a high impact on sensor performance offering unique features and increased surface area for biomolecular binding [17]. Nanostructures and their composites obtained from metal oxides of TiO_2_, CNT- TiO_2_, SnO_2_, ZrO_2_, etc., have been used in the design of glucose oxidase, cholesterol oxidase and other enzymatic biosensors [17–21]. ZnO, due to its multifunctional characteristics and ability to form anisotropic nanostructures, has shown potential for detection of biomolecules, drug delivery, in cancer treatment and for designing novel bioelectronics devices [22,23]. Structural stoichiometry is an important characteristic of a material that is utilized in creating tailored surfaces for suitable biosensor applications. ZnO has unique physical and chemical properties, in addition to structural quality, that make it a multifunctional material with specific surface selectivity tuned for biosensing applications. Furthermore, its single crystalline state favors the growth of nanostructured surfaces which offer unique nanomorphological and functional properties for biomolecule detection through capture probe immobilization. The stoichiometric composition with zinc interstitials and oxygen vacancies limit ZnO functionality in optical biosensors. However, due to its wide band gap and fast electron transfer kinetics ZnO is an excellent candidate material for designing sensors based on electrical/electrochemical transduction. The goal of this review is to provide a comprehensive understanding of ZnO characteristics and its application in design of biosensors which can help simulate new ideas and achievements in this field.

## Characteristics of ZnO

ZnO is a key technological material that has attracted profound research in the last decade due to its unique functional and nanomorphological properties. ZnO is an inorganic II–VI binary compound semiconductor with direct wide band gap of 3.37 eV in the near UV spectral region and large exciton binding energy of 60 meV at room temperature [24,25]. Such unique characteristics of ZnO have fascinated intensive interest among researchers in the scientific community to explore its use in versatile electronic and optoelectronic applications. As a wide band gap material, ZnO shows ability to withstand high temperature, larger electric fields, higher breakdown voltages and high power operation [24] and is a preferred transparent conducting metal oxide in semiconductor electronic applications. ZnO is naturally an n-type semiconductor because of the presence of zinc interstitials and oxygen vacancies in its crystal structure.

**Figure 2.**
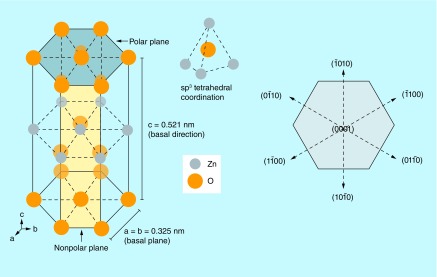
**A schematic representation of non-central symmetry in ZnO in its wurtzite structure highlighting the polar and nonpolar planes.** sp^3^ tetrahedral coordination in ZnO is also illustrated.

Though ZnO crystallizes in three different forms (i.e., zinc blende, wurtzite and rocksalt), the structure that is most thermodynamically stable under normal conditions of ambient pressure and temperature is its hexagonal wurtzite structure [25]. In wurtzite structure, as illustrated in Figure 2, crystallographic planes composed of Zn^2+^ and O^2-^ ions are stacked alternatively along the c-axis which confers noncentral symmetric structure to ZnO. This tetrahedral coordination between Zn^2+^ and O^2-^ ions gives rise to spontaneous polarity effects and induces piezoelectricity [26]. The layer-by-layer stacking of oppositely charged ions along the c-axis results in stable positively charged Zn-(0001) and negatively charged O-(0001) polar surfaces that results in different physical and chemical properties. Furthermore, availability of polar and nonpolar planes imparts strong electrical properties to ZnO which can easily be tuned and leveraged toward design of ZnO-based electrical biosensors.

Aside from causing the inherent polarity in the ZnO crystal, the tetrahedral coordination is an indicator of sp^3^ covalent bonding. However, the Zn–O bond also possesses very strong ionic character, which arises from large difference in electronegativity between zinc and oxygen atom. Thus, ZnO lies on the borderline between being classed as a covalent and ionic compound, with an ionicity of f_i_ = 0.616 on the Phillips ionicity scale [27]. Furthermore, the different relaxation energies of the polar surfaces favor the anisotropic growth of ZnO nanostructures with properties different from that of its bulk counterparts [28,29]. Transducer surfaces such as these are beneficial for biosensing because they can improve charge transfer-based biomolecular binding on select surfaces with high selectivity as well as enhance sensitivity of target biomarker detection.

## Suitability of ZnO as a material for biosensing

In recent years, ZnO thin films and nanostructures have shown great potential as a suitable material for binding of biological molecules. From a technological viewpoint, ZnO is an excellent candidate material for designing biosensor applications due to the ease of surface engineered terminations, high isoelectric point (IEP), fabricating their nanostructures at low temperatures and biocompatibility along with high electron mobility [30,31]. Physically and chemically tailored nanostructured ZnO surfaces can enhance sensitivity for detection by taking advantage of the smaller size, high surface area and electrical transport occurring at the nanostructured interfaces. The electrical transport properties in ZnO nanostructures will strongly depend on the crystal structure, surface polarity, etc., which can be tuned. The high ionic strength and pH stability make ZnO an ideal candidate for working with biological fluids [30]. ZnO possesses an IEP of 9.5 much higher than the IEP of most biomolecules and hence provides unique matrix for immobilization of biomolecules. At physiological pH, biomolecules are negatively charged due to a lower IEP and thus can be easily immobilized on positively charged ZnO through strong electrostatic interactions. This phenomenon is important to be considered during the selection of transduction material in design of biosensor devices.

Both ionic and semiconducting characteristics of ZnO can help provide the desired sensitivity for detecting target biomarkers at ultralow concentrations. Also, this particular metal oxide can be grown as nanostructures with current volume scalable manufacturing techniques and at low cost of ownership [32,33]. Therefore, it is then possible to integrate such an electrical biosensor using ZnO in a portable and inexpensive microelectronic device, which is necessary for POC diagnostics. The ability to tightly control the width, length and density of ZnO nanostructures allows for enhanced detection through physical confinement of biomolecules. Precise control over manufacturing conditions allows for greater selectivity by engineering the polarity of the ZnO surface. Studies also show that the performance metrics of ZnO-based biosensor for target biomarker detection can be enhanced by doping or creating complex hybrid composite matrixes, combining the excellent characteristics of ZnO and one other organic/inorganic material [34,35].

Researchers have studied an assortment of ZnO nanostructures for biosensing applications since their dimensions are comparable to the size of target biomolecules and thus allows for detection from low sample volume. ZnO nanostructures based on their dimensionality and nanomorphological characteristics offer opportunities for design of integrated biosensors for real-time sensing applications [36]. Such nanostructured ZnO biosensors in contact with the physiological fluid experience changes in their inherent electrical conductivity upon biomolecular binding which can be measured to demonstrate target biomarker detection. Tunable electrical properties in addition to ease of nanostructure formation can help achieve label-free detection through direct charge transfer mechanisms. The above-mentioned characteristics have been exploited to build ZnO-based analytical devices that operate under different configurations of electrical/electrochemical methods to demonstrate sensing of various biomarkers (such as glucose, urea, lactic acid and cardiac troponins, among others). In the following sections, this article provides a comprehensive review about methods for synthesis of ZnO nanostructures, functionalization of its surface and underlying mechanism for disease detection based on charge perturbations that occur at nanostructured ZnO sensing surface and sample medium.

## Synthesis of ZnO nanostructures

**Figure 3.**
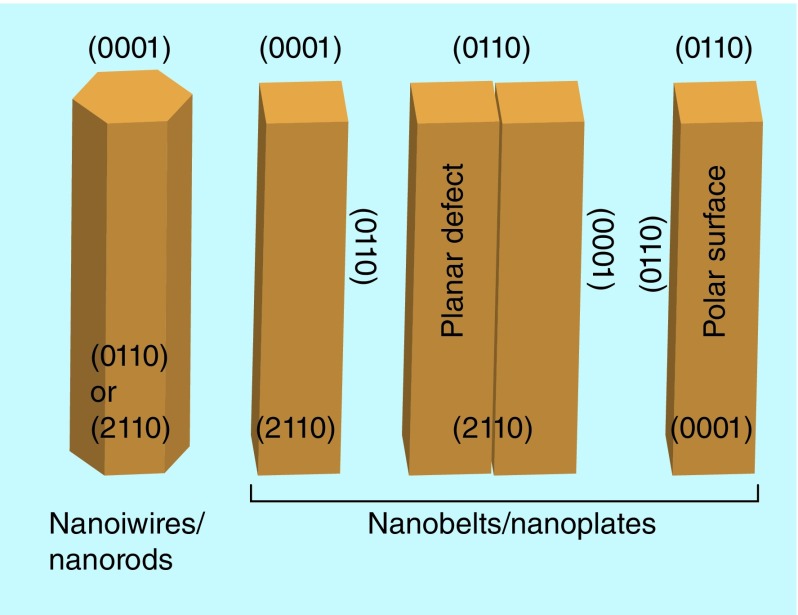
**Growth morphologies of ZnO nanostructures.** Adapted with permission from [29].

A diverse group of novel ZnO structures can be grown by tuning the growth rates in one of its three fast growing directions – (2110), (0110) and (0001) [29]. Thin films of ZnO can be formed on both crystalline and amorphous substrates by a wide variety of synthesis techniques. The different surface states and surface terminations are the main reason for the anisotropic growth in ZnO [37]. It has a very strong tendency to grow with its c-axis perpendicular to the grown substrate material due to the higher surface energy. Studies have demonstrated (0001) as the fastest growing direction for the formation of hexagonal wurtzite structured ZnO nanowire or nanorods whereas growth along other (21 10) or (0110) and its equivalent directions results in formation of nanobelts, nanoribbons or nanocombs [29], as illustrated in Figure 3.

The two main nanofabrication methods for fabrication of nanomaterials can be classified as: top-down (i.e., creation of nanostructures through step-by-step reduction of a bulk piece of material [38]) and bottom-up (i.e., starting the synthesis from the elements at the atomic scale to achieve desired growth of nanostructures through rearrangement and assembly [39,40]) synthesis methods [40]. However, bottom-up approach is a promising and preferred strategy to build ZnO structures at the nanoscale dimensions in the most efficient way possible. Nanostructures of ZnO using bottom-up approaches can be synthesized using both vapor phase and liquid phase methods [38,41]. The structure and morphology of nanostructures depend highly on the process conditions under which they are synthesized [38,41–44].

### Vapor phase synthesis methods

Vapor phase synthesis methods such as physical vapor deposition, chemical vapor deposition, vapor-solid or vapor-liquid-solid, and other epitaxial growth methods yield the best quality ZnO nanostructures [41]. ZnO nanostructures obtained by these methods involve decomposition of ZnO source material followed by condensation of vapor phase Zn^2+^ and O^2-^ on to the substrate. Nanostructure synthesis is normally carried out in a gaseous environment at temperatures ranging from 500 to 1500°C in closed chambers [38,43]. Growth of ZnO nanostructures in the above-mentioned techniques require precise control over the process conditions like temperature, pressure, flow rate, carrier gas concentrations or reagents, time and mainly the lattice parameters of the substrate [38,45]. Though these techniques offer the best quality nanostructures, a major requirement is the lattice matching of the substrate, which, if failed, may result in poor sample uniformity and different properties of nanostructures. These techniques require a highly controlled environment and sophisticated and expensive equipment for perfect and aligned growth of ZnO nanostructures [39]. Thus, synthesis of ZnO nanostructures with vapor phase techniques suffers from high cost and scalability issues [41,46]. The other shortcoming of these techniques is their poor amenability to flexible substrates due to the high processing temperatures.

Vapor phase process generally involves reduction of ZnO inside a reaction chamber. Growth of ZnO nanowires has been demonstrated by reducing a mixture of ZnO powder and graphite in Argon plasma followed by condensation of Zn vapor on Au-coated substrates [47]. Studies show that in such processes, thin films of metal act as a catalyst and the dimensions of nanostructure formation can be controlled by varying the thickness of the deposited metal layer. Jacobs *et al*. have investigated formation of ZnO thin films using RF magnetron sputtering under Argon plasma both in presence and absence of oxygen flow [48]. The ratio of zinc vapor pressure to oxygen vapor pressure controls the morphology of ZnO and thus induces defects in the crystal structure which, in turn, controls the electrical characteristics of the film. Due to the availability of a large number of synthesis techniques, the conditions and growth mechanism for the preparation of ZnO nanostructures vary across research groups [47,49–52].

### Liquid phase synthesis methods

Liquid phase or wet chemical synthesis methods are simple, scalable, cost-effective and requires a very simple equipment [38,43] and are the most widely used methods. Liquid phase methods like mechanochemcial process, sol-gel synthesis and solvothermal/hydrothermal processes involve the decomposition of zinc salts in an aqueous or organic chemical bath at low temperatures [41,43,44]. However, this nonconventional method is a two-step process that requires the formation of seed layer that acts as nucleation sites for the uniform and aligned growth of nanostructures on the substrate and the growth of nanostructures on the seed layer. The growth rate and uniformity of nucleation and thus, the physical and chemical property of ZnO nanostructures can be controlled by adjusting the growth parameters or by introducing additives [46,53,54]. These methods are amenable to flexible substrates as lattice matching is not a concern and is more controllable than vapor phase methods. Hence, wet chemical methods allow the growth of ZnO nanostructures directly on any organic substrate.

Vayssieres *et al*. were the first to successfully demonstrate synthesis of ZnO microtubes through chemical dissolution and aging process [55] and ZnO nanowires/nanorods from aqueous chemical solutions [56]. Ever since, different structures of ZnO both in micro- and nano-scale with controlled properties have been synthesized by several research groups [22,37,43–45,55–57]. In chemical processes, minimization of free energy of the underlying chemical reactions motivates the formation of ZnO nanostructures. ZnO does not normally hydrolyze under acidic conditions and since O^2-^ formation is from precursor salts rather than the solvent itself, an alkaline or aqueous environment is essential for decomposition of Zn salts. Nucleation begins when the chemical bath is supersaturated with ZnO nuclei, that is, when Zn^2+^ and O^2-^ ion concentrations in the solution have reached their critical value of saturation [58–60]. Thus the ZnO growth in the solution can be divided into two stages – ZnO nucleation and crystalline growth of ZnO nuclei. The main reactions that are involved in the growth of ZnO are summarized in the following chemical equations (Equations 1–4).
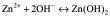


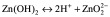


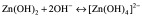


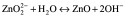



The type of zinc hydroxyl product formed depends on the concentration of Zn^2+^ ions as well as the thermodynamic equilibrium state of the chemical bath [44,61]. When ZnO nuclei is formed in the aqueous chemical bath, it adds a ZnO moiety to the polar surface. The ratio of zinc hydroxyl species formed over time is high and leads to faster growth along the polar (0001) face experiencing higher surface energy thus resulting in the formation of 1D ZnO nanostructures. Growth along (0001) face is favored as it is thermodynamically less stable than the well-ordered and packed nonpolar facets [60].

A myriad of ZnO nanostructures can be obtained by addition of additives such as citrate ions [62], ethylenediamine [63] or polyethylenimine which acts as capping agents that inhibit the growth along orthogonal or transverse directions [43]. Amino groups (-NH_2_) have high density of positive charges under normal growth conditions and wide range of pH which get adsorbed due to electrostatic attractions on nonpolar facets. On the contrary, citrate ions hinder the growth along the basal plane hindering the growth along the c-axis due to their negative charge density. While the former growth condition results in formation of nanostructures such as nanorods or nanowires or nanotubes, the latter results in formation of plate-like structures such as nanobelts, nanoribbons, etc. Zhang *et al*. have demonstrated the growth of oriented and hierarchical ZnO nanostructures with subsequent addition of two organic structure-directing agents – citrate ions and diaminopropane [63].

## Surface functionalization of ZnO

ZnO nanostructure surfaces obtained by the above described synthesis methods should be immobilized with a capture probe for detection of target biomarker. Sensitivity and selectivity of detection strongly depend on the stability of the capture probe immobilized on the ZnO nanostructure surfaces. Capture probe immobilization can be obtained either via covalent functionalization with organic molecules known as cross-linker or through physical adsorption. High IEP of ZnO facilitates physical adsorption of low IEP enzymes such as glucose oxidase, cholesterol oxidase, etc., on ZnO nanostructures through attractive electrostatic force. On the other hand, immobilization of capture probes such as antibodies for biosensing purposes rely on chemical binding with cross-linker molecules.

**Figure 4.**
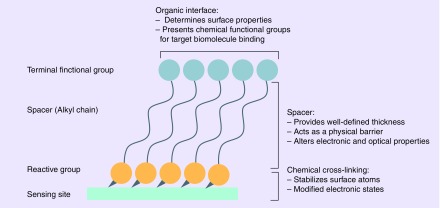
**Illustration of covalent functionalization of sensing platform with a cross-linker molecule.** Adapted with permission from [65].

Surface functionalization with cross-linkers results in formation of a highly oriented, ordered and packed organic layer on sensing the surface that provides selective surface sites for binding of biorecognition element. A cross-linker molecule is characterized by a reactive head group that covalently attaches to the sensing surface, an alkyl chain that acts as a spacer and a terminal functional group, usually NHS or NH_2_ ends, that facilitates interaction with amino acid residues [64,65]. A schematic representation of functionalization of any sensing site with cross-linker is shown in Figure 4. ZnO due to its structural inhomogeneities such as zinc interstitials and oxygen vacancies exhibits different binding affinities for various functional groups [66]. Several functional groups such as thiol, carboxyl and phosphonic acid groups have been investigated as surface linkers for binding to ZnO nanoparticles and thin films [67,68]. Very few studies have been directed toward evaluating binding efficacy on nanostructures such as nanorods and nanowires. Several factors including bond-stability, position of functional groups, pH, presence/absence of amine groups for interaction with antibody, surface charge, etc., play key role in selection of linker molecules [64]. Hence, it is important to develop a highly specific and sensitive biosensor with reliable surface chemistry for immobilization of biorecognition element while leveraging the material characteristics.

Silanization of ZnO surfaces with 3-aminopropyltriethoxysilane (APTES) toward biomarker detection has been demonstrated by few research groups [69–71]. Though surface modifications with siloxanes provide excellent hydrolytic stability, they formed a thin continuous monolayer which increased steric hindrance between biomolecules, and as a result, tailored charge transfer attributes would be lost. This led to reduced selectivity and subsequently reduced sensitivity toward specific detection of cardiac biomarker as demonstrated by Munje *et al*. [69]. Extensive characterization of thiol-based cross-linkers for the formation of self-assembled monolayers on gold substrates led to studies with ZnO as well. Modification of ZnO surfaces with thiol linkers for biosensing applications has been investigated by our group [48,69,72–74]. Although thiol modifications showed enhanced biosensing performance, extensive characterization of its binding nature was much needed to understand its reliability and stability. Surface terminations in ZnO may either result in formation of S-O-Zn or S-Zn linkages which greatly affect the bond enthalpy and, hence, the coverage. High density of surface functionalization on ZnO surfaces on the other hand can be achieved by phosphonic acid modifiers. Modifications with these linkers have shown to be reliable and can bind to ZnO surfaces in one of its three chemical binding modes [67,75]. The availability of three binding modes further gives space to control charge transfer rates at the electrode/electrolyte interface.

## Electrical characteristics of ZnO

The oppositely charged Zn^2+^ and O^2-^ ions stacked along the polar planes impart strong electrical properties to ZnO which can easily be tuned. As a consequence of direct and wide band gap semiconductor, ZnO has the ability to withstand high temperature and larger electric fields, higher breakdown voltages and high power operation [24]. N-type conductivity in ZnO arises from the presence of zinc interstitials and oxygen vacancies (both the levels are occupied by electron pairs) [76]. Synthesis of p-type ZnO remains a challenge due to self-compensation caused by the native defects [15].

The electrical properties of ZnO are strongly influenced by the adsorption of oxygen species from ambient air, which in turn controls the carrier concentration. Either physisorption (does not result in any electronic transfer process) or chemisorption (oxygen species now acts as electron acceptor sites) changes the overall resistance of ZnO material [77]. It is also known that due to low carrier concentration, ZnO in its purest form has high resistivity that has high significance in memristor and many gas sensing applications [78]. On the contrary, low resistivity of ZnO can be obtained by increasing the oxygen vacancies or by doping which again influences the electron mobility [79].

## ZnO nanostructured electrode/electrolyte interface

**Figure 5.**
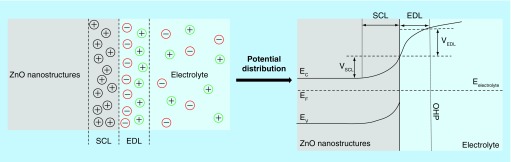
**Illustration of charge and potential distribution at ZnO electrode/electrolyte interface.** Adapted with permission from [80].

Charge transfer between the semiconductor and the electrolyte at the interface is key to the development of ZnO-based electrical/electrochemical biosensor. The lattice structure of material under study significantly influences the bulk and interface properties. Due to imperfections in crystal lattice which includes surface states and abrupt lattice terminations, different facets of crystal may come in contact with the electrolyte. This significantly alters the charge distribution at both ZnO electrode and electrolyte side [80]. Localized charge distribution within zinc oxide constitutes space charge layer (SCL), whose thickness is in order of few nanometers and is specific only to semiconducting electrodes [81]. This region is occupied by immobile charged impurities or trapped carriers or charge carriers of the semiconductor. Next region is the characteristic double layer formation that occurs at the interface of two phases. This region constituted by the adsorbed species, defects and impurities in the surface, solvated ions and chemically bonded molecules form the Helmholtz layer or the electrical double layer [81]. The electrical double layer consists of inner Helmholtz plane formed by adsorbed ions and solvent species and outer Helmholtz plane formed by the center charge of solvated ions. Extending into the bulk of the electrolyte is the third region constituted by concentration profile with excess of solvated ions that forms the diffuse layer also known as Gouy layer [81]. When an electric field is applied, the electrode is polarized and charges realign at the interface as described earlier resulting in potential distribution across these layers, as shown in Figure 5.

The majority of charge carriers in an n-type semiconductor are the electrons. So when ZnO nanostructured surface is brought in contact with the electrolyte, there occurs positive charge build up in SCL due to electron transfer from its conduction band to the electrolyte and negative charge build up in the electrolyte. When an electric field is applied, unlike metal the potential drop occurs across both SCL and HL, as shown in Figure 5 [82,83]. The gradient potential distribution occurring at SCL results in band edge pinning at E_C_ and E_V_, while constant potential is observed in the bulk of the semiconductor. Hence, it is extremely important to understand the mechanism of charge transfer and correlating them to the effects of biomolecular binding interactions is necessary for quantification of target biomarker concentrations. When an electric potential is applied to ZnO, the charge distribution occurring at the interface due to biomolecular binding events results in a potential drop between the electrode and electrolyte [72]. Modulation to surface potential can be measured as changes to threshold voltage, flat-band potential or capacitance based on device configuration and measurement modality. In the following section, we discuss applications of ZnO-based biosensors for disease identification and biomarker quantification.

## Cardiovascular disease diagnosis

A group of pathological disorders of the heart and blood vessels affecting not just the coronary artery but the entire arterial circulation is termed cardiovascular disease (CVD). The clinical manifestations of CVD include coronary heart disease, angina, myocardial infarction or heart attack, chronic kidney disease, stroke and carotid artery disease [84]. According to heart health statistics report by American Heart Association, globally about 17.3 million deaths per year occur due to CVD and is expected to rise to more than 23.6 million by 2030 [85]. Once every 40 s about 2150 Americans die due to CVD accounting for nearly 787,000 deaths each year in the USA alone [85]. Approximately 85.6 million Americans are affected annually by CVD – a major threat to mankind [86]. Though cardiac imaging and ECG indicate the functions of heart, it still cannot be treated as a standalone test for the diagnosis of CVD. During ischemic necrosis of heart muscles (a condition called as acute myocardial infarction [AMI]), most times ECG shows no signs of ST-segment elevation which results in discharge of patients without proper diagnosis. Studies show that testing the biological fluids for elevated concentrations of biomarkers specific to different stages of cardiac pathogenesis assist in successful diagnosis and therapeutic management of disease. Traditional assays such as ELISA have longer turnaround times that interfere with timely diagnosis. Therefore, a more sensitive and rapid platform that can reliably detect cardiac biomarkers in real time is important to improve diagnostic ability of clinicians.

Venous blood of the infarcted patients, or patients complaining of chest pain are sampled to test for different markers of AMI. Biomarkers which include proteins, enzymes or metabolites are expected to be released into bloodstream when cardiac muscles experience stress due to ischemia [87]. When the heart muscles are stressed due to AMI, certain substances called as biomarkers indicative of pathophysiological conditions are released into the blood stream [88,89]. Measurement of these biomarkers could help in successful diagnosis and prognosis of diseases. However, since CVD includes spectrum of disorder the cardiac biomarker under evaluation should meet specific criteria: specific and sensitive to a heart disorder; longer diagnostic window; early diagnosis – should be released into bloodstream quickly and; reliable. Therefore, determination of elevated concentrations of cardiac biomarkers in serum samples is critical in early management of AMI.

Detection of biomarkers in particular concentration ranges is related to AMI diagnosis and risk stratification of cardiac injury. Enzymes such as aspartate transaminase, lactate dehydrogenase and creatine kinase (CK) are conventional cytoplasmic markers indicative of cardiac tissue injury [87,90]. Aspartate transaminase is not specific for cardiac injury as it is also found in liver cells and are also indicative of liver damage. Similarly, the presence of lactate dehydrogenase in body tissue renders it nonspecific. CK and its isoenzyme – CK-MB (CK-myoglobin) though recommended by WHO are not 100% specific to cardiac injury [91,92]. Also these markers offer shorter diagnostic window as they return to normal levels within 24–48 h [93]. Tissue necrosis is irreversible if not identified within shorter treatment window. A sensitive and specific biochemical marker for AMI diagnosis was satisfied with the discovery of cardiac Troponins in 1980s [92]. Troponins have high specificity and sensitivity to cardiac injury and are found in bloodstream for up to 14 days [91,94]. Tissue-specificity and sensitivity of cardiac isoforms (cardiac troponin-T [cTnT] and cardiac troponin-I [cTnI]) favor the developments of assays for obtaining diagnostic and prognostic information from serum samples of patients with coronary syndrome [95,96]. Though several assays and biosensors for cTns are commercially available, efforts are still underway to reduce the nonspecificity and increase the sensitivity to enable early diagnosis. According to National Academy of Clinical Biochemistry recommendations, clinical testing of serum samples for cardiac biomarkers should be made available with turnaround times less than 1 h in all cases [97]. Rapid turnaround time can be achieved through development of POC devices that meet these unmet challenges. Table 1 summarizes the performance of ZnO-based biosensors for detection of cardiac biomarkers.

**Table 1.** **A summary of the electrical biosensors based on ZnO for detection of cardiac biomarkers.**

**Cardiac biomarker**	**ZnO nanostructures type**	**Detection format**	**Detection range**	**Lower detection limit**	**Ref.**
cTnT	Nanorods	Immunosensor	1 fg/ml to 100 ng/ml	100 fg/ml	[72]

cTnT	Nanorods	Immunosensor	1 fg/ml to 100 ng/ml	1 pg/ml	[74]

cTnI	Nanoparticles	Electrical	1 ng/ml to 10 μg/ml	2.19 ng/ml	[71]

cTnI	Nanoparticles	FET	1 ng/ml to 10 μg/ml	3.24 pg/ml	[72]

cTnT & cTnI	Nanorods	Immunosensor	100 fg/ml to 100 ng/ml	1 pg/ml	[73]

FET: xxx.

In one study, the authors developed a nanostructured sensing platform with ZnO nanorods to demonstrate detection of cTnT based on electrical/electrochemical transduction mechanism [72–74]. ZnO nanorods, prepared through a wet chemical-based hydrothermal synthesis method, were used as the immobilization matrix to capture antibodies. They demonstrate that the electrochemical behavior of ZnO nanobiosensor depends on crystalline structure, aspect ratio and density of ZnO nanorods. Authors show dependency of electrode robustness based on varied density of ZnO nanorods [72]. Tunable density was obtained by modulating the molar concentrations (i.e., 10, 25, 50, 75 and 100 mM) of precursor salts – hexamethylenetetramine and zinc nitrate hexahydrate (Zn(NO_3_)_2_.6H_2_O) dissolved in water. In c-axis-oriented growth, the polar planes can either be Zn-terminated or O-terminated. Under such conditions, the electrical characteristics of ZnO can be tuned by modifications to their surface, which can effectively be used for biomarker identification. In this study, results showed modulation in charge transfer and effective charge density with varied density of ZnO nanorods, a phenomenon attributed to variation to the number of zinc interstitials and oxygen vacancies. Also, each ZnO nanorod (prepared under 50 mM growth condition) at the sensing platform behaves as an individual nanoelectrode as it facilitated semi-infinite hemispherical diffusion of cardiac troponins to its surface. This behavior thus enhances the sensing ability of nanostructured ZnO electrode as opposed to sensing with nanotextured ZnO surfaces demonstrated previously by our group [48].

Detection capabilities of the developed nanostructured ZnO biosensor were tested with aliquots of spiked concentrations of cTnT in human serum. Detection was established based on changes in charge distribution at the ZnO electrode/human serum buffer interface. cTnT binding and subsequent polarization cause imbalance in surface electrochemical potential at the interface that was measured as changes to capacitive impedance through electrochemical impedance spectroscopy and Mott–Schottky. The authors show that binding events cause localized positive and negative charge distribution on ZnO (known as SCL) and electrolyte side (known as Helmholtz layer), respectively. Electroionic changes modulate capacitance at both these layers and, hence, demonstrate label-free and sensitive detection of cTnT with detection limit at 0.1 pg/ml using above-mentioned complementary techniques.

However, the essential requirement for potential application as a POC biosensor is the ability to work with low sample volume and function with low power which led to incorporation of the above design on flexible polyimide substrates [73,74]. Similarly, simultaneous detection of cTnT and cTnI could help in risk stratification of AMI. In this study, a multiplex ZnO-sensing platform comprising of two arrays of working and counter electrodes with a common reference was used to simultaneously detect and quantify cTnT and cTnI [73]. Cross-reactivity and nonspecificity of capture antibodies (α-cTnT and α-cTnI) were established with bovine serum albumin and its alternating cardiac isoforms, respectively. An output signal response much below established signal noise threshold (three-times background noise signal) was observed. Background signal noise was estimated with blank human serum treatment on antibody-treated ZnO biosensor. In another study, they demonstrate robustness of flexible nanostructured ZnO biosensor though cyclic bending experiments [74]. The researchers observe very minimal loss in sensing performance under tested strain conditions. The above results demonstrate feasibility of multiplexed detection of cTnT and cTnI in biological sample using complementary AC and DC techniques with high sensitivity and specificity with detection limit as low as 1 pg/ml.

Detection of cTnI using ZnO nanoparticle-based sensing platform has recently been demonstrated by several other research groups. Tan *et al*. developed an APTES-functionalized ZnO nanoparticle-based interdigitated electrodes biosensor that was found to detect cTnI biomarker [70]. Detection was achieved based on current changes that occur pre- and postbiomarker binding and demonstrated detection limit at 2.18 ng/ml. In another study, cTnI detection was established by monitoring the changes to drain current and threshold voltage using a ZnO nanoparticle-based field effect transistor biosensor in a pnp configuration [71]. Multiple layers of ZnO nanoparticles reduce the resistance of the n-type region which upon cTnI binding causes change in drain current under biased condition. Substrate-gate coupling in FET configuration increased the sensitivity of detection with limit down to 3.24 pg/ml. These studies open up new pathways toward development of ZnO-based POC biosensors for ultrasensitive and selective detection of cardiac biomarkers.

## Conclusion & future perspective

A key revolution in biosensor development is the integration of structures at the nanoscale to obtain enhanced analytical performance. Nanostructured sensing surfaces have opened up new horizons for biosensing applications. Further, biomolecule immobilization at these nanostructured surfaces can be achieved in a selective and oriented fashion without losing the biological activity of biomolecule. Sensor technologies with integrated nanostructures are becoming increasingly attractive due to their proven potential for development of portable and miniaturized POC biosensor for rapid biomarker detection.

Thin films and nanostructures of ZnO, due to their fascinating properties, can offer novel electrical and electrochemical properties. These properties can be exploited through surface engineering of ZnO for achieving high sensitivity and selectivity toward biomarker detection. In addition, scalable manufacturability of ZnO and tailored surface properties make it possible to fabricate ZnO biosensors with excellent detection capabilities. At the nanoscale, ZnO experiences fast electron transport kinetics and, hence, is sensitive to any physicochemical changes occurring at its surface even in presence of ultralow biomarker concentrations. Formation of a biorecognition layer on the surfaces of ZnO through covalent binding is a complex procedure which depends on surface topography and defects in ZnO crystal structure.

Conventional diagnoses for cardiovascular diseases have been focused on quantitative determination of its biomarkers in whole blood or heparinized plasma samples. Despite tremendous progress in developing POC diagnostic devices for cardiac disease diagnosis, a substantial number of devices lack methods for standardization, concerns with false positives if sample preparation is inadequate and need for catheter-based blood draw in most cases. Recently, studies have shown scientific evidence relating clinical information from saliva to cardiac biomarkers. Saliva, as a diagnostic fluid offers new directions in development of POC devices for cardiac disease diagnosis. However, design of ZnO-based platforms for diagnosis with saliva may be highly challenging due to their effect on salivary peroxidase enzyme as well in those patients treated for oral conditions.

Current efforts are directed toward understanding ZnO sensor architectures, which are focused on achieving low sample handling, high throughput and multiplexed detection. Although most ZnO biosensors discussed in this review meet the clinical need, translation of such devices into routine diagnostic practices is still a long way away. The ability to demonstrate sensitive and selective detection in natural biological buffer is extremely important for practical realization of these devices in the biosensor industry. Nonetheless, the current state-of-the-art technologies will be of great scientific value for further advancing ZnO biosensors performance for POC diagnostics applications.

**Executive summary**

- Cardiovascular disease is the leading cause of death worldwide. Development of low-cost and point-of-care diagnostic devices for disease identification is the need of the hour.
- ZnO exhibits multifunctional characteristics which favors sensitive and selective detection of target biomarkers.
- Nanostructures of ZnO can be fabricated using low-cost techniques. The structural morphology of grown structures is effectively controlled by various growth parameters.
- Rapid charge transport at ZnO electrode/electrolyte interfaces can be interpreted toward detection of target biomarkers.
